# Important aspects of conducting an interdisciplinary public preventive oral health project for children in areas with low socioeconomic status: staff perspective

**DOI:** 10.1186/s12903-020-01352-8

**Published:** 2020-12-17

**Authors:** C. Blomma, B. Krevers

**Affiliations:** 1 Public Dental Service Östergötland, and Centre for Medical Technology Assessment, Department of Health, Medicine and Caring Sciences, Linköping University, 581 83 Linköping, Sweden; 2 Centre for Medical Technology Assessment, Department of Health, Medicine and Caring Sciences, Linköping University, Linköping, Sweden

**Keywords:** Qualitative research, Caries, Family, Staff, Child health services, Maternal health, Promotion, Implementation, Stigmatization

## Abstract

**Background:**

To achieve good and equal oral health in children, interdisciplinary preventive oral health actions, directed toward disadvantaged areas, can be an important means. Staff play a crucial role in the implementation of these actions. The aim of the present study was to analyze circumstances of importance for conducting an interdisciplinary public preventive oral health project for children, directed toward parents in areas with low socioeconomic status from the interdisciplinary perspective of the involved staff.

**Method:**

The present study consisted of a qualitative content analysis with an inductive approach, based on interviews with interdisciplinary staff who had participated in a public preventive oral health project directed toward parents in areas with low socioeconomic status. The interviews were analyzed using text-driven analysis.

**Results:**

The main category concerned the staff members’ prerequisites and understanding necessary to perform their tasks in interdisciplinary public preventive oral health project. To have the right prerequisites and understanding regarding the oral health project enabled staff to be committed, able and willing to perform in it. Important aspects of this are to have knowledge, motivation and to experience a supportive professional context, to have good leadership and for certain resources to fulfilled. A crucial aspect was to reach the targeted mothers.

**Conclusions:**

For interdisciplinary cooperation in preventive oral health care to be achieved, it is essential for the involved disciplines and professions to embrace a common view on the project’s aim, their duties, and oral health, from the leadership to the individual level. Staff require competent leadership but also allocated time and adapted method support to be successful in this context. When allocating preventive health actions directed at low SES areas, it is important to acknowledge the risk of stigmatization and for staff to understand that families might be facing social challenges that prevent them from taking part in health-promoting actions. An important conclusion is that to be able to reach people, it is important for both those who design preventive programs for oral health and the staff who administer them to have sufficient knowledge about the target group.

## Background

### Caries and caries prevention

Oral health is part of overall health and is important for children’s wellbeing and quality of life [1, 2]. Caries is the most prevalent oral health disease and early experiences predispose an individual to continued disease progression later in life [1–4]. Thus, it is important not only to cure but most of all to prevent caries. Caries can be prevented by a combination of actions at home, and at both a professional and a community level by prevention, early detection and intervention [1, 2, 5, 6]. Since small children are completely dependent on their parents to supply them with good oral habits and to provide them with oral health care, research has proposed that caries prevention should be directed toward pregnant women, small children and their parents [7–11]. Maternal and child health caregivers can play an important role in preventive oral health care [11, 12], and interdisciplinary preventive oral health actions have been successful in decreasing caries prevalence in children [13, 14].

### Caries prevalence and socioeconomic status

In Sweden, preventive oral health efforts have contributed to a considerable improvement in children’s oral health over the last few decades, but the improvement is unequally distributed [15, 16]. Children living in areas with low socioeconomic status (SES) have poorer overall health and more caries than children from areas with higher SES [17]. Studies from across the world show the same pattern [18, 19]. In Sweden, overall 16 percent of 3- to 6-year-old children have experience of dental caries, but children living in the lowest SES areas have a five times higher risk than children living in areas with the highest SES [17]. This is despite the fact that oral health care is free of charge for children and young adults in Sweden, up to the age of 23, as is maternal and child health care (until the age of 18 years).

In order to reduce differences in oral health between socioeconomic groups, it has been suggested that efforts should be interdisciplinary, aimed at addressing the risk factors that are common to oral and overall health, and extended in proportion to the needs of the population, so-called universal proportionalism [12, 20, 21].

### Settings and the interdisciplinary public preventive oral health project

An interdisciplinary public preventive oral health project that was conducted in a multicultural area in Sweden with low SES between 2013 and 2018 [22]. The objective of the health project was to evaluate a program for improved oral health in young children living in the area and to create a model for health promotion, caries prevention and reducing the differences in caries prevalence in these children compared with other socioeconomic groups. The project was conducted as a scientific prospective intervention study. The target group consisted of pregnant women (referred to as mothers in the paper) living in an area with low SES in a city in the southeast of Sweden. (The design of the project is presented in Additional file 1: Appendix 1). The oral health project consisted of:

- a questionnaire to assess caries risk of the family
- a caries prevention program consisting of several different actions, depending on the assessed caries risk of the family, and including oral health-promoting talks using the motivational interviewing (MI) technique as a method, and
- interdisciplinary cooperation between Maternal Healthcare Services (MHS), Child Healthcare Services (CHS) and Public Dental Services (PDS).

The project was introduced by project managers (heads of all three disciplines and specialists in pediatric dentistry and public dental health). The oral health-promoting talks were given at the PDS, from prebirth until the child turned 3 years old, by PDS staff who had been educated by a psychologist as oral health-promoting moderators in the motivational interviewing technique. Interpreters were used when needed, the project material was translated into ten different languages and designed in consultation with health-educated communicators with relevant cultural knowledge. Because of difficulties in recruiting individuals of the target group to the project, the recruitment phase was prolonged from the initial 1 year to 2 years, and 64 mothers completed the project [22]. A project evaluation was performed. The data presented here emanates from the project evaluation and this study is part of a comprehensive evaluation of the public oral health project.

The staff play a crucial role in the implementation of preventive oral health actions [23]. It is therefore important to ascertain what circumstances are significant for staff in the conducting of a project of this kind in the given setting. There is still a lack of research on cross-disciplinary and inter-professional implementation of oral health programs.

### Aim

The aim of the present study was to analyze what circumstances are important for conducting an interdisciplinary public preventive oral health project for children directed toward parents in areas with low socioeconomic status, from the interdisciplinary perspective of the involved staff.

## Method

The study was designed as a qualitative content analysis, based on interviews. Content analysis is used for making inferences from texts to the context of their use. A text-driven approach was employed to explore the interviews. This is an inductive point of entry to the content analysis that searches a text for its meaning. In this process the analysts also must be aware of their own conceptual contribution as well as what the text was intended to express based on those interviewed [24]. The consolidated criteria for reporting qualitative research (COREQ) was followed when reporting this study [25].

### Recruitment and interviewees

The recruitment was strategic in the sense that the inclusion criterion was having worked in the project, and representatives from all the various participating disciplines and professions were included in order to access a variety of experiences and perspectives. We recruited the interviewees from three different settings: Maternal Healthcare Services (MHS), Child Healthcare Services (CHS) and Public Dental Services (PDS). Those who took part in the interviews were: Four midwives and one care administrator from MHS, four child health nurses from CHS, and three dental hygienists and one dental nurse from PDS. For the CHS and PDS, this was a total survey of all the staff working in the project. For MHS staff, a combination of strategic (different professions) and convenience sampling (availability on the day of the interview) was used to include an equal number of interviewees as from the other disciplines, selected from the 21 MHS staff who were working in the project.

Potential interviewees were contacted by telephone and asked to participate in the study by the interviewer. Prior to the interview, all interviewees received written information about the study, its aim and how data would be stored and used. The interviewees were assured of confidentiality and that participation was voluntary. The written information was sent by email, and before the interview took place the interviewee verified verbally that they had received it. Verbal informed consent was obtained from all interviewees prior to the interview.

### Data collection

A semi-structured interview guide was developed and used (presented in Additional file 2: Appendix 2) [26]. It consisted of questions about the interviewees’ role and assignment within the project, what feedback they had received, what contacts they had within the project, their views on and perceptions of the project and also what they considered to be others’ (colleagues’ and mothers’) perceptions of the project.

The interviews took place as the prevention project was about to be finalized when recruitment at the MHC was completed and only single visits to the CHS remained.

All interviews were conducted in Swedish by an external interviewer (AA) with a background in dentistry but without any relation to the prevention project or the interviewees. The location for each interview was the interviewee’s workplace, in order to make the logistics easy and to reduce travelling time for them. The interviews (range from 10 to 29 min, average 16 min) were recorded and transcribed verbatim.

### Analysis

A content analysis with text-driven approach was performed to analyze the meaning of the data, which consisted of the 13 interviews with the staff transcribed to text [24]. To obtain an overview of the content, the interviews were read through several times by the author (CB) and a colleague (KA), the further analysis was drafted by the first author (CB). The text was divided into textual units, consisting of words and sentences that were related to each other through their content and context, corresponding to the aim of the study. These textual units were then condensed into shorter meaning units, while still preserving the core meaning. These condensed textual units were labeled with codes that briefly described the content. The analysis was then discussed with the co-author (BK). The further process of data analysis was a collaborative effort between the authors (CB and BK), in which codes that were perceived as referring to the same issue were gathered into categories and subcategories. In cases of disagreement, the authors revisited the data and aimed to reach consensus after further discussions. From these categories, an overall summarizing main category was composed. The process was not linear but meant that the authors went back and forth between data and analysis. This was done to make sure that the analysis was based on the interview data. Quotes presented in this paper were translated into English by the author (CB) and reviewed and revised by a professional native English-speaking reviewer. Thereafter again reviewed by the authors (CB and BK).

Author CB (female) is a doctoral student and a dentist with several years’ experience of working with families in low SES areas. Author BK (female) has extensive experience of qualitative research and a background in health care.

## Ethics

The ethical principles for medical research involving human subjects according to the Declaration of Helsinki were followed concerning the interviewees’ right to informed consent and to withdraw their participation, and confidentiality. All data was anonymized and stored such that it was inaccessible to unauthorized people. This study does not include research involving interventions on the part of participants and therefore does not require ethical approval according to §3 or §4 [27].

## Results

The main category concerned the staff members’ prerequisites and understanding necessary to perform their tasks in this interdisciplinary public preventive oral health project. To have the right prerequisites and to understand their role and assignment regarding oral health prevention enabled the staff to be committed, able and willing to be engaged in the project. The main category was related to the five categories presented below. Staff required *knowledge* and *motivation* and to work in a *supportive professional context*, in terms of having understanding colleagues. Staff also needed to perceive good and sufficient *leadership and resources* to be able to commit and perform. A crucial aspect of the performance of the project was *reaching the mothers*. In order to do so, it was important that both the involved disciplines and the parents achieved a common understanding about the oral health project and its benefits to ensure that they were committed to the project. Each of the five main categories above contain two to three subcategories highlighting various aspects, presented in Table 1.

**Table 1:** Overview of the results: main theme with associated main categories and subcategories

Prerequisites and understanding necessary to perform	1. Knowledge	1a. Information
		1b. Competence
		1c. Learning
	2. Motivation	2a. Previous experience
		2b. Expectations
		2c. Attitudes
	3. Supportive professional context	3a. Comprehension
		3b. Professional responsibility
	4. Resources and leadership	4a. Support
		4b. Time
		4c. Leadership
	5. Reaching the mothers	5a. To communicate
		5b. To motivate
		5c. Conception of the target group

The results also show that the different professions had different views on their competence and what was their own responsibility when it came to preventive measures for oral health. This could have consequences for their engagement in the project. However, the professional groups shared the experience of the circumstances that were important for conducting an interprofessional oral health prevention project, as shown in the following results.

### Knowledge

#### 1a. Information

In order to facilitate the staff members’ execution of the project, it was important for them to receive information concerning why and how it was to be conducted, its aims, their tasks, feedback about the current situation and who to turn to with questions. Who communicated the project to staff, and how they did so, was also important. If information was received from secondhand sources, e.g. colleagues, and either only verbally or only in writing, this was considered a barrier because it made the information unclear. Staff appreciated being given general information about the project, mediated by the project managers and disseminated at the beginning of the project. This was also helpful in the process of comprehending the project and why each discipline was to be a part of it. Meanwhile, it was also important to provide general information for staff who started work later on, when the project was already under way. These new staff members complained about the lack of information, and that they had an imperfect understanding of the project, which reduced their expectations and motivation to conduct the work.

“Are we to get yet another thing?” Because we have it quite tough, with a lot of information for the patient at our first appointment. But when [project managers] came and presented and we saw pictures of the children, none of us was doubtful. Of course we should be a part of it. (5 MHS)

Regular meetings with project managers and colleagues were perceived as facilitating the project because questions could be resolved quickly, information and feedback could be given and such meetings facilitated personal contact with others working in the project. Written information from these meeting was appreciated as a way to stay informed.

#### 1b. Competence

The project work was facilitated if staff had knowledge and/or experience of oral health, research, motivational interviewing and working with patients from other cultures and speaking other languages. Interviewees who had these skills felt competent when performing their assignments and it facilitated their work within the project. It was important for staff to have sufficient knowledge about oral health, research methods and the project to motivate the mothers to join it, and to be able to answer supplementary questions. Lack of these competencies could lead staff to accept mothers’ refusal to participate rather than encouraging them to do so, because they could not provide additional information explaining to mothers what it meant to participate or what the benefits would be.

This written consent, many thought it was strange. “What does that mean?” And in the beginning I couldn’t really answer it either, what it meant, so then it was even weirder. (12 CHS)

Education in motivational interviewing was valuable, not only for the project but also due to its usefulness in interviewees’ ordinary work. When the moderators carried out the education together, there was potential for group cohesion. For staff to feel competent in the method, it was important to practice the skills frequently and to do so immediately after the education.

#### 1c. Learning

Collective learning across disciplines was developed during the project, both in meetings and during the conducting of the project, for example, when using the written project material in communication with the mothers. Staff from the different disciplines learned about how the others worked, facilitating both the project’s work and the staff’s ordinary work alongside it. Hence, staff were able to respond to questions from the mothers about each other’s disciplines. Working and learning across disciplines developed a mutual message about oral health and diet, which facilitated communication with the families.

We’ve had joint meetings and reviewed what we provide as advice regarding diet and dental care, etc. So, we’ve been able to provide roughly the same information to families as dentists and dental nurses. (11 CHS)

The project and the screening questionnaire were perceived as reminders that helped the staff to talk frequently with the families about their children’s oral health starting early in the children’s lives. Interviewees hoped that this awareness of oral health promotion would linger within their discipline even after the study, and that perhaps the project would result in a continued and/or extended collaboration between dental health care, nutritionists, MHS and CHS in the future.

### Motivation

#### 2a. Previous experience

Previous experience of unequal oral health in young children or experience of working with public health questions were factors that positively affected staff members’ motivation to conduct the project. Interviewees also described their previous experience of the target group of mothers as challenging to reach but rewarding when they succeeded. This contributed to a positive attitude and an enthusiastic view of the assignment, which was important in the conducting of the project.

I thought it was positive to try to reach these families as early as possible. And oral health is a problem, we see a lot of children who suffer who actually have caries in their teeth and have had them removed when they are 1½ years old. (10 CHS)

#### 2b. Expectations

Having positive expectations of the project facilitated the staff’s motivation to participate in, and to conduct, the work. Such expectations were manifested in curiosity, eagerness to help, anticipation and hopefulness about a positive outcome in both the short term (e.g. that the mothers would come to their appointments) and the long term (e.g. that it was a good idea to reach these families earlier in terms of caries prevention and that fewer children would get caries henceforth). Knowledge and understanding about the aims of the project, to improve oral health and to evaluate a new way of working with this issue, were factors affecting staffs ‘expectations positively. Interviewees who described an absence of expectations did not know what outcomes to expect and lacked information about the project.

So I jumped at it, because I thought it sounded interesting and you want so much and we can’t go home to everyone and brush the children’s teeth in the evening, it’s not possible, so you want to try to reach out as much as you can (2 PDS)

#### 2c. Attitudes

Various aspects of the project could affect staff members’ attitudes towards it, as well as their motivation to be part of it. Staff who considered oral health to be part of children’s overall health, and thus something that concerned all the involved disciplines, were motivated to be part the project. Diet is considered central to health promotion in all three disciplines, which is why all of them were considered appropriate to be a part of the project.

Diet, pregnancy, dentistry, they really belong together, everything. Because we work a lot with the diet, that they shouldn’t gain too much weight and what they eat and drink, and that’s pretty much the same as the dental care project. (7 MHS)

Interviewees described a range of attitudes concerning their involvement in the project, varying from feeling responsible for planning its implementation within their own discipline, to merely that of executing a task, such as distributing questionnaires to the mothers. Although there was a lot of work involved in both the planning and delivery of the project, it was considered valuable and positive to be part of both.

Understanding the background to the project and why it was being undertaken could positively affect the attitudes of the participating staff towards it and increase their motivation in their project-related work. Regular information and feedback about the project, provided by committed project managers, was a way to achieve this. Interviewees described how their commitment waned as the study was extended over time but that their motivation could be increased by positive feedback regarding the project. But knowing about the aims of the project, for example, and considering them important did not always have the same positive effect on staff members’ attitudes towards participating in the project.

It is a positive goal you’re working towards, absolutely, but it’s more when you have to see our [discipline] part in it as well. (6 MHS)

### Supportive professional context

The professional context concerns colleagues, both those participating in the project and others.

#### 3a. Comprehension

Colleagues’ comprehension about the caries situation of the children in the area contributed to a positive view of what the project could accomplish. Having a colleague with whom to discuss issues, give feedback and share experiences and knowledge with was a relief, supportive and gratifying. Lack of comprehension about why it was important for their discipline to participate was a reason why colleagues did not commit to the project and therefore did not prioritize it, or (fully) perform their tasks. Interviewees described having colleagues who did not commit to the project or perform well in it as surprising, unexpected and a barrier to conducting the work.

Colleagues expressed views that the project would fail, that the study group was hard work, hard to reach and would not come to their appointments, and frustration about filling in for staff to cover ordinary routines while they participated in the project. Colleagues’ views could be positively affected by information and feedback about the improvements achieved through the project, enhancing collegial comprehension about the project.

But now when it has been a while and we have actually reached some mothers and after all you see some results, then they [colleagues] think it´s funny (3 PDS)

#### 3b. Professional responsibility

Colleagues views upon their professional responsibility concerning oral health in relation to their profession was important for their commitment to the project and collegial support they provided. For example although colleagues may have agreed that the aim of the project was positive, or that it was strategic to work with their discipline in order to reach these families early, they did not find the project applicable to their discipline because they did not work within the field covered by it. Interviewees described colleagues stating that oral health was outside their working field, that it was unimportant in relation to other tasks and that the project took time away from their ordinary duties.

Everyone thinks that it’s a good topic, that it’s a good place to recruit study patients, but it may not feel applicable in our discipline and, as I said, we don’t work within that field. (8 MHS)

Colleagues who were committed to the project thought the interdisciplinary approach was positive in that it enabled a holistic view of the mothers’ situation and helped to forge cooperation between the disciplines.

It’s very positive that you also look after the whole person, the person and that you don’t just concentrate on the pregnancy, many have seen this as positive. (9 MHS)

### Resources and leadership

#### 4a. Support

Developed routines, IT systems and colleagues were resources that could act in a supportive manner during the project when they were in place and working well.

Producing and establishing routines that worked well for the various tasks performed by each participating discipline were supportive and facilitating for the staff in the work of the project. An example of this was the identification of the target group and the distribution of questionnaires to the personnel at MHS, which was accomplished by an administrative officer, thus relieving the project staff. In contrast, the lack of appropriate routines was described as a barrier in the conducting of the project. For example, unclear routines could result in not all the questionnaires being distributed as they should, or the answers not being returned, which created more work.

Dissimilar and non-functioning IT systems in the different disciplines caused problems in the project. Interviewees requested integrated interdisciplinary personnel, with general knowledge of and access to the different IT systems, in order to facilitate activities such as booking appointments.

Appointments were booked that we didn’t know about, so expectant mothers might come in, bringing a note with them saying that they have a time for an oral health promotion talk, and then it’s not booked onto our timetables. (1PDS)

#### 4b. Time

Having sufficient time allocated to perform project tasks was an important resource for the staff that meant they experienced less stress and had time for dialogue with the mothers. Whilst a lack of time created difficulties for staff to commit to the project or to give mothers sufficient information for them to commit. This dilemma was particularly problematic with mothers who needed an interpreter or were illiterate. Due to time constraints, the project sometimes had to wait, and occasionally it was not executed at all. Colleagues could be helpful in terms of having someone to share the workload and create more time to fulfill one’s duties within the project.

We get a lot of assignments, things we have to do, it´s added all the time but the time [to perform them] is not increased nor the personnel (CHS 13)

Interviewees also described how, despite time constraints, they tried to squeeze in as much as possible during their appointments, including the project, reminding themselves of its purpose when it was challenging to get everything done.

#### 4c. Leadership

Leadership concerned both the local management of each discipline and the project managers and affected the staff members’ ability to commit and to be able to conduct the project. Whether duties within the project were carried out or not was perceived as a question of leadership, or the lack of it.

A big difficulty has been the communication with a discipline. It seems that some staff actually don’t care about the project, they don’t distribute the questionnaires.// So, it’s those difficulties, it feels like some don’t take this seriously, but at the same time, I feel, it’s a matter of management; if you’ve been assigned a duty, you should actually do it. (4 PDS)

Regular meetings with project managers, being able to make requests, being involved in decision-making about one’s duties in the project and being listened to were also issues that facilitated the interviewees’ ability to conduct the project. For example, when participants requested information material or announced that they did not have sufficient time, it was appreciated if their statements resulted in new, adapted working material, an additional colleague or altered routines.

### Reaching the mothers

#### 5a. To communicate

The interviewees stated that it was difficult for the staff to communicate and for the mothers to comprehend what the project and research were about. They perceived that mothers who understood the meaning of the project thought it was interesting and positive, and one explanation given for this was that parents want what is best for their children. Mothers who did not understand often refused to participate. Several mothers did not know what the appointment at the PDS was for, or perceived it to be an appointment for an examination of their own mouth and teeth, and when they realized it was “just” for talking, they thought the meeting was pointless.

Communicating with illiterate mothers or mothers who spoke a different language, even when using an interpreter, was described as a major barrier and a time-consuming aspect of the project and of reaching the mothers. Telephone interpreting was considered especially time consuming, difficult and non-spontaneous. Even though staff asked interpreters to translate verbatim during the oral health promoting talks, they did not always do so, perhaps because they were not used to the method (motivational interviewing). The sex of the interpreter could be important, because some mothers did not want to speak freely to a male interpreter.

It’s the language barrier that’s difficult, even sometimes if you have an interpreter, it can be difficult. (12 CHS)

The written information produced for the project and the fact that it was distinct, concise and translated into many different languages was valuable for the personnel when they communicated the project to the mothers. The closed-ended questions were perceived as facilitating for the target group when filling out the questionnaires. However, the questions concerning level of education and eating habits were considered difficult because of cultural differences related to eating and difficulties in translating level of education to Swedish levels. There were interviewees who stated that, in order to get the questionnaires properly filled out, the mothers needed a lot of support from them.

The written consent, which included written information about the study, could be difficult for the mothers to read and understand, and for them to comprehend what participating in the research would entail for them. In particular, mothers with a different native language or from other cultures may have declined participation for this reason.

Motivational interviewing was considered a good method to use in oral health-promoting talks with the target group because the open questions made it possible to get more out of the conversation with the mothers, compared with ordinary one-way communication/information. However, communicating with motivational interviewing could be unfamiliar to the mothers, and interviewees pointed out that perhaps they were more used to taking directives from health staff rather than talking and reflecting with them about health advice. Motivational interviewing in combination with interpreters was considered difficult in the oral health-promoting talks.

The method of interdisciplinary working across professional boundaries, and even integrated at the same workplace, was considered supportive for vulnerable mothers in terms of communicating oral health including giving them access to professional knowledge and guidance in dental care.

#### 5b. To motivate

Mothers’ experiences of oral health problems, or a lack of contact with oral health services for themselves, were factors that positively affected their motivation to participate in the project. Interviewees perceived that the mothers who received oral health-promoting talks were grateful and pleased to have received new information. It was easier to commit the mothers to continuing oral health-promoting talks after their child was born than during pregnancy. An explanation given for this was that the topic seemed more real to the mothers after their child was born. Another circumstance affecting the mothers’ motivation was whether they felt they had the ability to participate or not. Lacking a residence permit, for example, could affect life conditions and the ability to commit to the project, even though it was perceived as valuable.

Some [mothers] have thought “No, what is this?” and “I don’t want to participate” or “I may not be able to join right now" if they haven’t received a residence permit to stay in Sweden. (3 PDS)

#### 5c. Conception of the target group

Interviewees described several conceptions of the target group that affected their ability to reach this group in order to commit them to the project.

The target group of mothers and their children was described as a disadvantaged group that needs support, requires more information and is hard to reach and communicate with, perhaps especially the mothers who need it most. Furthermore, they are often late or do not show up for their appointments, including during the project, even though they themselves choose the time and the staff put a lot of effort into reminding them. Interviewees described how mothers would say that they were going to come to their appointment, that they were positive about the project, that they would fill out the questionnaire and return it, but then did not. The interviewees expressed a feeling of frustration and sadness related to this, and also because the mothers did not understand the benefits of participating, and the personnel only wanted to help them. Staff perceived that sometimes the target group did not see any point in having pregnancy or oral health checks, and that perhaps oral health was not important to them as long as there was no pain. Perhaps oral health was not as important to the mothers as other problems, such as disease, violence, social problems or how to put food on the table.

The group is generally hard to reach, because there are many who are late for their appointments, who fail to come to many appointments and are often in need of an interpreter. And sometimes they can’t see the point in having their pregnancy checks, and even less being able to answer questions based on research and the Biobank Act etc. because they’re from cultures where this perhaps is not something you talk about in daily practice. (8 MHS)

Interviewees described how mothers in the target group said they felt stigmatized by the offer to participate in the oral health-promoting project because of where they lived. They had felt offended and construed the invitation to participate as an accusation of having bad oral health or not taking care of themselves and their oral health. Therefore, they did not want to be a part of the project.

## Discussion

This is a qualitative interview study involving interdisciplinary staff. It analyzed their perspectives on the circumstances that are important for conducting an interdisciplinary public preventive oral health project directed towards parents in areas of low SES. The main category concerned the staff members’ prerequisites and understanding necessary to perform their tasks in interdisciplinary public preventive oral health project. Possessing the right prerequisites and understanding regarding the oral health project enabled staff to be committed, able and willing to perform in it. Important aspects of this was knowledge and motivation, as were practical resources, leadership and a supportive professional context, which concerned the interviewees’ colleagues and their comprehension about the project and views on their professional responsibility. The most crucial factor for staff to be able to conduct an interdisciplinary public preventive oral health project directed towards parents in areas with low SES was reaching the mothers to commit them to project.

This paper adds more knowledge about interdisciplinary cooperation and the necessity for staff to have an understanding about oral health prevention to commit to such cooperation, and how this understanding is generated. It demonstrates the importance of the professional context and interactions between staff, both within and between disciplines and professions, for the implementation of a project of this kind. Most importantly, it elucidates various aspects that are important in reaching the target group. All these circumstances eventually affect the health outcomes of the project’s target group.

Our study emphasized that knowledge and motivation are essential for staff to commit and perform in interdisciplinary cooperation, which is also well known in implementation research [23]. In this context, they need information about the project, but above all they need knowledge about oral health and oral health needs in the staff’s professional context. Furthermore, it is also essential for them to be able to commit the mothers to such an initiative. Ongoing information about the public health issues that are the target of the interdisciplinary actions, along with feedback on their progress, can enhance staff members’ motivation to conduct their tasks within the project. This is especially important for staff who started work after the project had begun and in projects that are ongoing for an extended period of time, to ensure staff members’ knowledge and motivation to understand, commit and perform.

Our results showed that different aspects of oral health’s relation to overall health can affect staff members’ attitudes to whether oral health prevention is part of their professional role and professional responsibility or not. Although preventive oral health measures were not a given part of the professional role for the involved professionals, there were colleagues who were convinced that this was an important part of their work. These personal and/or professional aspects were crucial for staff members’ motivation to commit and perform, and for colleagues to do the same. Consequently, they are important for whether tasks are performed with interdisciplinary oral health cooperation. Recent research also shows that, in order for preventive actions to be undertaken, it is important that the staff who are to implement them perceive them as part of their professional role [28]. Our results support the idea that a common view on these aspects, in and between disciplines and professions, is fundamental in an interdisciplinary cooperative project of this kind. Since oral health is important for overall health, and oral health risk factors are also common to several other chronic diseases, an interdisciplinary and interprofessional approach to oral and general preventive health measures could benefit public health work. This has also been suggested in earlier research [9, 29–31]. For this to happen, primary health-care providers need knowledge about the impact of oral health on general health and well-being [32]. The present study has demonstrated an interdisciplinary collaboration of this induced learning across disciplines, which increases knowledge about the involved disciplines and about oral health. This facilitates communication with the target group and enhances their interest in interdisciplinary work. The integration of oral health at the educational level for all health-care professionals would increase knowledge about oral health and generate a common view on public health. In this way, it would provide conditions for oral health issues to be addressed in meetings with all public healthcare professionals, hence leading to a better quality of public health work.

The results revealed appropriate leadership and different resources, such as time, to be important for staff to commit and perform their tasks. These prerequisites have also been identified in earlier research on implementation [23, 33]. In the present context, in areas with low SES, time allocated is an especially important prerequisite for overcoming cultural and linguistic communication barriers to create understanding and commitment within the target group.

In the results, *leadership* appears as the prerequisite for ensuring that the project is properly conducted. This is well known from the literature and other studies [23, 34, 35]. In the present context, it means that it was important for leaders to clarify what the staff should do and provide important resources for them to be able to do it, such as time and support. Furthermore, a vital aspect of this leadership can be the promotion of a common view on oral and general health amongst the different professions involved which can have a positive impact on the professional context and interdisciplinary cooperation.

The results of the present study identified several important circumstances in the process of reaching the mothers living in areas with low SES in order to commit them to the preventive oral health-promotion project. An important finding was the difficulties in reaching parents who were considered by the staff to be in greatest need of support. These parents could be very hard to reach and it was difficult for them to comprehend the project. For example, a written consent form could prevent the target group from participating in the project because they felt insecure about signing a paper they did not understand or mistrusted what it meant to sign this paper. This is in line with other research, which has also found that this group is difficult to reach, and previous discussions about how signed informed consent might contribute to a low participation rate among families with low incomes [36–38]. This highlights the necessity of assigning enough time to design and deliver appropriate information and support that is comprehensible and makes sense to the target group. This is an important quest and more research is needed to improve the understanding of how people from other cultures and backgrounds can be reached in our society with the aim of enhancing their health.

One conception of why it was challenging to reach the target group concerned the mothers’ difficult social situation, which was considered to prevent them from committing to a prevention project of this kind. The fact that experiences of an overload in daily life, due to demands related to daily living and survival, can mean that parents fail to take their children to the dentist because health-promotion is low priority for them, has also been shown by other researchers [39]. On the one hand, the conception of the target group as disadvantaged and in need of support acted as a motivation for staff in their work on the project. On the other hand, this conception caused stigmatization in the target group and among the mothers being identified by the project. It has been suggested that stigmatization of families who utilize projects, and mistrust of the staff who provide them, may be common [39, 40]. Non-judgmental staff and good relationships between staff and patients, built on trust and respect, are vital in order to reach families [39–41]. Our results suggest that it is important to recognize the risk of stigmatization when allocating preventive health actions toward low-SES areas. Therefore, staff members need to understand the emotions this may inflict on the individuals targeted by this intervention, and to ensure that they are not made into victims or blamed.

Another aspect of reaching the mothers was that the timing of the oral health intervention, starting at the beginning of pregnancy, was not optimal for the recruitment of mothers to the project. This barrier could be overcome by including community residents in the planning of preventive actions in their community, to ensure that the design and content are adequate and appropriate for them and their needs. This would hopefully increase residents’ motivation and improve the acceptability of preventive actions among the community when such actions are implemented [23]. This may be particularly important in socially disadvantaged communities, where trust in the health-care system and the use of preventive oral healthcare are lower [42–44].

This study suggests that, even though staff considered motivational interviewing to be a facilitating method to communicate information about preventive health to the target group, there were several cultural, linguistic and practical barriers that limited communication using motivational interviewing in this context. These barriers may help to explain why studies of motivational interviewing as a method for behavioral change and caries prevention have shown varying results in recent research, suggesting that motivational interviewing is not as efficient in high-risk populations as elsewhere [45–47]. To overcome these barriers, it is important that interpreters are familiar with the method and able to point out cultural differences, for example when motivational interviewing is inappropriate for the target group [48]. This also emphasizes the need for research to explore how people with different cultures and experiences from the majority perceive and think about health care and health.

### Considerations

One potential weakness of this study is that the interview guide was originally developed with another aim, which was to evaluate the project. However, the interview data was rich and well suited to the aim of the present study. The fact that neither of the authors conducted the interviews may reduce the risk of anything that was not transcribed verbatim being taken into consideration in the analysis. Furthermore, the analysis was conducted by two researchers with different experiences and from different disciplines, CB being a dentist and doctoral student and BK an experienced qualitative senior researcher. This may reduce the risk of misinterpretation and the influence of preunderstandings.

Although the project was ongoing for several years and the interviews were conducted at the end, we consider them to have provided information about the entire period, and by that point the participants were also more experienced. The fact that the participants came from different professions and disciplines means that they were able to provide comprehensive information about the conducting of interdisciplinary projects of this kind and context.

The number of interviewees in the study can be considered quite limited. However, this was because there was a limited number of employees working in the project, and in two of the three disciplines everyone was recruited who met the criteria for our study. For the third, an equal number of participants as from the first two was included in order to balance the amount of data material from each discipline. Qualitative content analysis emphasizes the breadth and variations in the data [24]. The resulting text from the interviews is rich in data and expresses a variety of both positive and negative aspects, which enhances the credibility of the data. We have only commented briefly in the results on disciplinary differences because the small number of individuals from each discipline prevented us from drawing sweeping conclusions about differences between professions in a credible way.

The transferability of the present results to other interdisciplinary oral health promotion programs in other parts of the world may vary, depending on how dental care is organized and financed. However, we consider the present results to be applicable to the development of interdisciplinary cooperation projects in similar settings, as the primary results concern intra- and interpersonal aspects of the staff members’ understanding, as well as their willingness and ability to reach the target group. We consider that our findings are also supported by previous studies in other settings [23, 28, 33–39].

### Implications

This study implies that, in the planning of interdisciplinary oral health promotion:

- leaders and managers need to ensure that staff in different disciplines develop a common view of oral health as an essential aspect of overall health. This is an important starting point for staff to gain an understanding of the project’s goals and benefits and their individual responsibilities and obligations towards the target group,
- leaders and managers in the involved disciplines need to ensure that important prerequisites for preventive oral health care, such as clear and accessible leadership, resources in terms of time and technological support, and working methods and routines, are in place for frontline staff,
- both the designers of preventive programs for oral health and the frontline staff have sufficient knowledge of the target group and have insight into the conditions under which families facing social challenges can participate in health promotion measures,
- acknowledge the risk of stigmatization in the target group when allocating preventive health-care actions in areas of low SES.

## Conclusions

It is a great challenge to reach families living in areas of low SES with preventive oral health interventions, and this challenge is an interdisciplinary issue. It is essential in interdisciplinary cooperation that the involved disciplines and professions embrace a common view on the project’s aim, their duties, and oral health, from the leadership to the individual level. Staff require competent leadership but also allocated time and adapted method support to be successful in this context. When allocating preventive health actions directed at low SES areas, it is important to acknowledge the risk of stigmatization and for staff to understand that families might be facing social challenges that prevent them from taking part in health-promoting actions. An important conclusion is that to be able to reach people, it is important for both those who design preventive programs for oral health and the staff who administer them to have sufficient knowledge about the target group . Future research ought to further explore different perspectives on and experiences of preventive oral health care among parents in areas of low SES to improve knowledge of how to reach families in this context with appropriate preventive oral health programs. And to examine how different professions perceive their responsibility for oral health as a part of their public health prevention assignment.

## Supplementary Information

**Additional file 1: Appendix 1**. The design of the interdisciplinary public preventive oral health project.

**Additional file 2: Appendix 2**. Interview guide. The semi-structured interview guide used in the interviews.

## Data Availability

The data used and analyzed during the current study is available from the corresponding author.

## Abbreviations

SES: Socioeconomic status
MHS: Maternal Healthcare Services
CHS: Child Healthcare Services
PDS: Public Dental Services
