# Supplementary material for: Important aspects of conducting an interdisciplinary public preventive oral health project for children in areas with low socioeconomic status: staff perspective
Source: BMC Oral Health. 2020 Dec 17;20:362. doi: 10.1186/s12903-020-01352-8 (PMC7745486; doi:10.1186/s12903-020-01352-8)
Supplement: Supplementary file 1 — Additional file 1: Appendix 1. The design of the interdisciplinary public preventive oral health project. [file 12903_2020_1352_MOESM1_ESM.docx]

Appendix 1. The design of the interdisciplinary public preventive oral health project

| Pre-birth |  | **INTERVENTION GROUP**  Pregnant women in specified low SES areas  n=175 |  | **CONTROL GROUP**  Other pregnant women (intervention group excluded) in other areas of the municipality during study period n=1325 |
| --- | --- | --- | --- | --- |
|  |  | First visit to MHS:  Questionnaire 1 for caries risk assessment  Appointment for an oral health promoting talk |  | First visit to MHS:  Questionnaire 1 for caries risk assessment |
|  |  | One or more oral health promoting talks at the public dental services (PDS), according to assessed caries risk |  | Ordinary  routines in maternal healthcare |
| Birth |  | Individual caries prevention program at the PDS, according to assessed ECC risk |  | Ordinary  routines in child healthcare |
|  |  |  |  |  |
| 1 month |  | Questionnaire 2 at CHS |  | Questionnaire 2 by postalmail |
| 12 months |  | Oral health promoting talk Questionnaire 3 by postalmail |  | Questionnaire 3 by postalmail |
| 18 months |  | Oral examination and oral health promoting talk |  | Ordinary  routines in child healthcare |
| 24-36 months |  | Individual caries prevention program at the PDS, according to assessed ECC risk |  | Ordinary  routines in child healthcare |

|  | Ordinary routines |
| --- | --- |
|  | Events in oral health program |

MHS= Maternal healthcare services

PDS= Public dental services

CHS= Child health services
