# Supplementary material for: Important aspects of conducting an interdisciplinary public preventive oral health project for children in areas with low socioeconomic status: staff perspective
Source: BMC Oral Health. 2020 Dec 17;20:362. doi: 10.1186/s12903-020-01352-8 (PMC7745486; doi:10.1186/s12903-020-01352-8)
Supplement: Supplementary file 2 — Additional file 2: Appendix 2. Interview guide. The semi-structured interview guide used in the interviews. [file 12903_2020_1352_MOESM2_ESM.docx]

**Appendix 2.**

Interview guide

| Area | Question | Follow up question |
| --- | --- | --- |
| The interviewee | Can you please tell us what function you have at your workplace? |  |
| The assignment | Describe how you perceived your assignment in the public preventive oral health project |  |
|  | How did you get this assignment / who gave you the assignment? | What was your opinion of this assignment? |
|  | Was the assignment clear? | Would it have been possible to increase clarity? |
|  |  | Were the aims of the assignment clearly described? |
|  | What were the facilitating factors? | Did you feel you had the right skills? |
|  |  | What skills were required for your task? |
|  |  | Did you feel well prepared after the education in MI? |
|  |  | Do you think MI is a good method for the oral health promotion talks? |
|  | What were the impeding factors? | Did you feel you had sufficient knowledge? |
|  |  | Did you have enough time for the assignment? |
|  |  | Where there anything you needed more time for? |
|  | What were your expectations on the project? | If any, have they been met? |
| Perceptions | What views do your colleagues seem to have about their assignment in the project? |  |
|  | What views seems the study group to have about the project? | Positive/ negative views? |
|  | How did the study group seem to perceive MI as a method for oral health promoting talks? | Positive/ negative views? |
|  | Do you perceive that the questionnaire is easy to complete? | What was good, what was bad? |
|  | Do you perceive that the study group thought the questionnaire was easy to complete? | What was good, what was bad? |
|  | Do you perceive that the invitation to the project is easy to understand? | What was good, what was bad? |
|  | Do you perceive that the study group thought the invitation to the project was easy to understand? | What was good, what was bad? |
| Feedback | Do you feel that you have received feedback during the assignment? | If not, would you have liked that? |
|  |  | How in that case? |
| Contacts/ security | Have you known who to contact in case of any uncertainty/ question? |  |
